# Supplementary material for: Predicting visual function by interpreting a neuronal wiring diagram
Source: Nature. 2024 Oct 2;634(8032):113–23. doi: 10.1038/s41586-024-07953-5 (PMC11446822; doi:10.1038/s41586-024-07953-5)
Supplement: Supplementary file 6 — CRF and ERF predictions for individual TmY4 and TmY9 cells. Analogous to Supplementary Data 3, but for TmY target types. Shown are the top four monosynaptic pathways, the strongest pathway passing through each of the top ten intermediary types (ranking from Extended Data Fig. 7), and the trisynaptic pathway Tm1–TmY–Dm3–TmY (see the section entitled Prediction of spatial normalization). [file 41586_2024_7953_MOESM6_ESM.zip › DataS4/TmY9q/720575940637621360.pdf]

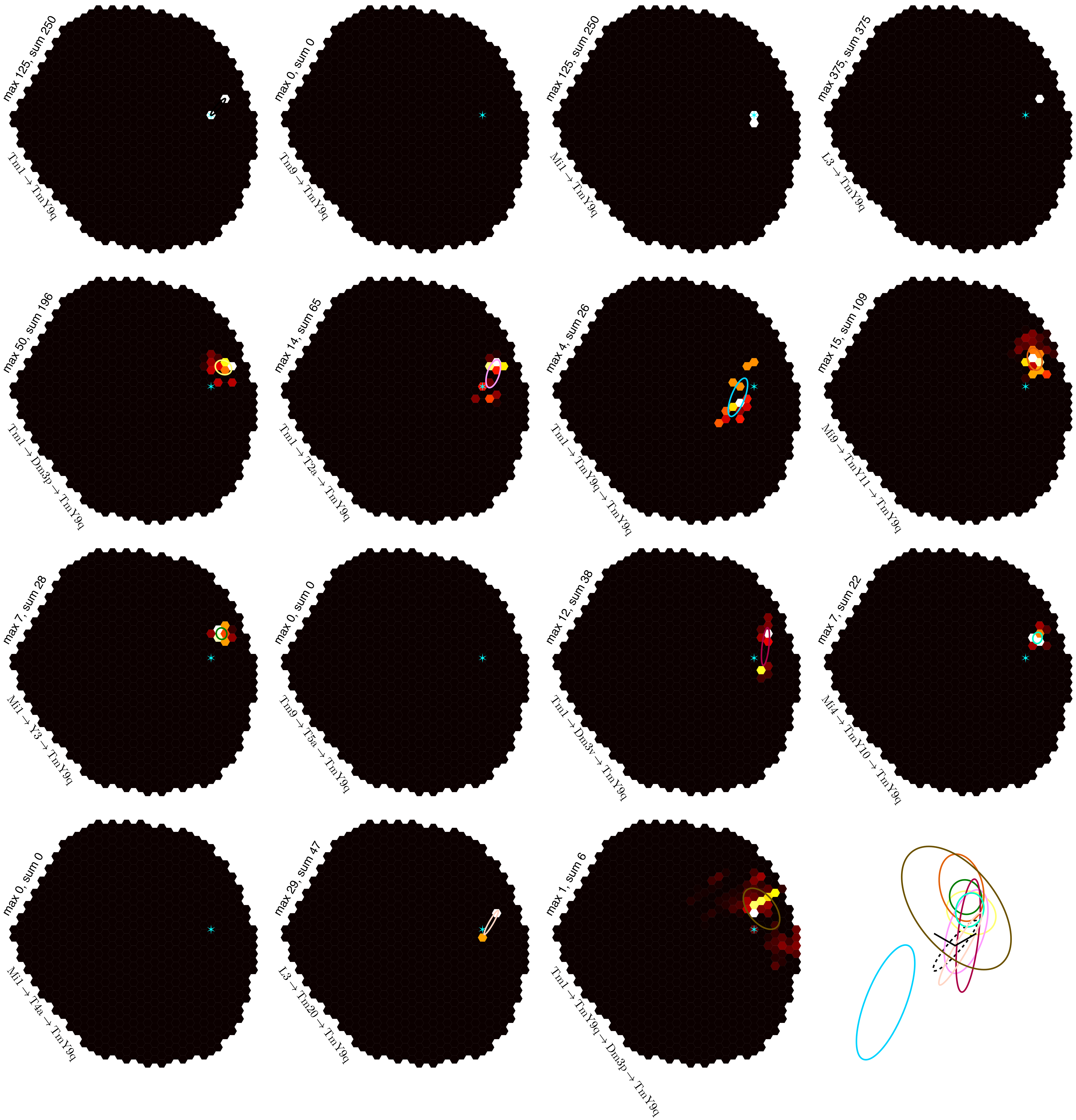
max 125, sum 250
Tm1 → TmY9q
max 0, sum 0
Tm9 → TmY9q
max 125, sum 250
Mi1 → TmY9q
max 375, sum 375
L3 → TmY9q
max 50, sum 196
Tm1 → Dm3p → TmY9q
max 14, sum 65
Tm1 → T2a → TmY9q
max 4, sum 26
Tm1 → TmY9q → TmY9q
max 15, sum 109
Mi9 → TmY11 → TmY9q
max 7, sum 28
Mi1 → Y3 → TmY9q
max 0, sum 0
Tm9 → T5a → TmY9q
max 12, sum 38
Tm1 → Dm3v → TmY9q
max 7, sum 22
Mi4 → TmY10 → TmY9q
max 0, sum 0
Mi1 → T4a → TmY9q
max 29, sum 47
L3 → Tm20 → TmY9q
max 1, sum 6
Tm1 → TmY9q → Dm3p → TmY9q
